# *Lonicerae Japonicae* Flos Extract Promotes Sleep in Sleep-Deprived and Lipopolysaccharide-Challenged Mice

**DOI:** 10.3389/fnins.2022.848588

**Published:** 2022-04-12

**Authors:** Ruifang Hua, Yan Ding, Xiaolong Liu, Bingxuan Niu, Xinfeng Chen, Jingjing Zhang, Kerui Liu, Pei Yang, Xiaofei Zhu, Jintao Xue, Hui Wang

**Affiliations:** ^1^Henan Key Laboratory of Immunology and Targeted Drugs, School of Laboratory Medicine, Xinxiang Medical University, Xinxiang, China; ^2^College of Pharmacy, Xinxiang Medical University, Xinxiang, China; ^3^Chinese Institute for Brain Research, Beijing, China

**Keywords:** *Lonicerae Japonicae* Flos, Chinese herbal medicine, sleep, immune system, lipopolysaccharide, proinflammatory cytokines, hippocampus, medial prefrontal cortex

## Abstract

*Lonicerae Japonicae* Flos (LJF) is commonly used in Chinese herbal medicines and exhibits anti-viral, anti-oxidative, and anti-inflammatory properties. The reciprocal relationship between sleep, the immune system and the central nervous system is well-established in the animal models. In this study, we used the mouse model to analyze the beneficial effects of the LJF on the dysregulated sleep-wakefulness cycle in response to acute sleep deprivation and lipopolysaccharide (LPS)-induced inflammation and the potential underlying mechanisms. Polysomnography data showed that LJF increased the time spent in non-rapid eye movement (NREM) sleep during the day under basal conditions. Furthermore, latency to sleep was reduced and the time spent in rapid eye movement (REM) sleep was increased during recovery from acute sleep deprivation. Furthermore, LJF-treated mice showed increased REM sleep and altered electroencephalogram (EEG) power spectrum in response to intra-peritoneal injection of LPS. LJF significantly reduced the levels of proinflammatory cytokines such as IL-6, TNF-α, and IL-1β in the blood serum as well as hippocampus, and medial prefrontal cortex (mPFC) tissues in the LPS-challenged mice by inhibiting microglial activation. Moreover, LJF increased the time spent in REM sleep in the LPS-challenged mice compared to the control mice. These results suggested that LJF stimulated the sleep drive in response to acute sleep deprivation and LPS-induced inflammation, thereby increasing REM sleep for recovery and neuroprotection. In conclusion, our findings demonstrate that the clinical potential of LJF in treating sleep disorders related to sleep deprivation and neuro-inflammation.

## Introduction

*Lonicerae Japonicae* Flos (LJF) is also called *Jin Yin Hua* in the China Pharmacopoeia and is commonly used in traditional Chinese medicine (TCM), healthy Chinese foods and beverages (Jintao et al., 2021; Wu et al., 2021). The flowers of LJF show antiviral, anti-inflammation, anti-tumor, antioxidant, anti-radiation, and immune regulatory properties (Gao et al., 2018; Ge et al., 2019; Li et al., 2020). In China, LJF is an essential ingredient of the Lianhua Qingwen capsule (LQC), a patent TCM formula for preventing infection in coronavirus disease 2019 (COVID-19) (Hu et al., 2020; Xia et al., 2020; Lee D. et al., 2021; Zhao et al., 2021). A combination of TCM drugs such as LQC and Western antiviral medicines showed beneficial effects in control of COVID-19. TCM formulas significantly alleviate the symptoms and progression of viral infections. LJF is one of the most commonly used TCM in the treatment of COVID-19 (Luo et al., 2020). Therefore, there is an urgent need to investigate the biological functions of LJF.

To date, more than 300 compounds from the LJF extract including volatile oils, organic acids, and flavonoids have been identified (Tang et al., 2021). Chlorogenic acid and luteolin are used as quality control standards for the characterization of medical materials (Yuan et al., 2014). LJF extract suppressed inflammation by downregulating the expression of inflammatory cytokines (Lin H. W. et al., 2021; Liu C. et al., 2021). Meanwhile, the central nervous system coordinately regulates inflammation and sleep (Irwin and Opp, 2017). Among the known active ingredients in the LJF extract, chlorogenic acid and luteolin are associated with increased sleep time and decreased sleep latency (Park et al., 2017; Kim et al., 2019). However, the mechanisms by which the LJF extract regulates sleep homeostasis have not been investigated previously.

Adequate sleep is essential for the physical and psychological health of the individuals and is regulated by the circadian and homeostatic processes in different species (Pan and Kastin, 2017; Scammell et al., 2017). Wakefulness is characterized by high levels of sensory awareness to various environmental stimuli, whereas, non-rapid eye movement (NREM) sleep and rapid eye movement (REM) sleep represent two different states of sleep architecture that are regulated by homeostatic and circadian mechanisms (Brown et al., 2012). Sleep homeostasis is regulated by sleep pressure, which is induced by both internal and external stimuli. Sleep architecture is significantly altered in sleep disorders, mood disorders, and infectious diseases (Besedovsky et al., 2019; Irwin, 2019; Atrooz and Salim, 2020). Epidemiological studies have shown that sleep deficiency is associated with inflammation, neuropsychiatric diseases, and multi-organ injury (Leproult et al., 2014; Irwin et al., 2016; Atrooz and Salim, 2020; Feng et al., 2020; Ramos-Lopez et al., 2021; Zhang et al., 2021).

The central nervous system coordinately regulates inflammation and sleep through multiple mechanisms (Irwin and Opp, 2017). Lipopolysaccharide (LPS) is a key component of the gram-negative bacteria that induces inflammation by activating massive production of pro-inflammatory cytokines such as interleukin (IL)-1β, IL-6, and tumor necrosis factor (TNF)-α. Elevated levels of pro-inflammatory cytokines in the blood and brain trigger immune defense mechanisms and adaptive behaviors, including sleep. Sleep disturbances contribute to the risk of inflammation and are associated with increasing levels of circulating IL-6 (Irwin et al., 2016). TNF-α regulates sleep by acting on several established sleep regulatory neural circuits and alters the synaptic plasticity of the nervous system (Rockstrom et al., 2018).

The circadian rhythm and sleep homeostasis are regulated by multiple brain regions including the prefrontal cortex, thalamus, hippocampus, and complex neural network. Normal sleep is essential for the proper regulation of memory and mood (Girardeau et al., 2017; Sawangjit et al., 2018). The medial prefrontal cortex (mPFC) regulates sleep-wake cycles and is visualized by specific EEG bands. The mPFC also regulates emotions and cognitive functions in coordination with the hippocampus (Lou et al., 2020; Varela and Wilson, 2020; Dimitrov et al., 2021). Hippocampus plays a key role in cognition, mood, and memory (Sierra et al., 2014; Lee J. H. et al., 2021). Neurons in the cortex and hippocampus are essential components of sleep regulation as visualized by EEG oscillations in these regions (Levenstein et al., 2019). These data demonstrate the clinical significance of the relationship between brain, sleep and immunity. Therefore, the aim of this study was to investigate the effects of LJF on the sleep-wakefulness cycle under basal, sleep-deprived, and LPS-induced inflammatory conditions.

## Materials and Methods

### Animals

Male C57BL/6J mice (8–12 week old; weight: 20–25 g) were purchased from the Beijing Vital River Laboratory Animal Technology Co., Ltd. (Beijing, China). They were housed in the mouse facility at the Xinxiang Medical University under standard housing conditions that included constant temperature (22 ± 2°C), humidity (55 ± 5%), and 12 h light/dark cycle [lights on at zeitgeber time (ZT) 0 or 07:00 a.m.] with *ad libitum* access to food and water. The Henan Provincial Animal Care and Use Committee approved all the animal protocols. The animal experiments followed the principle of minimizing the number of animals and alleviating pain and were performed in accordance with the experimental guidelines approved by the Animal Experimentation Ethics Committee of the Xinxiang Medical University, China.

### Preparation of the Ethanol Extract of *Lonicerae Japonicae* Flos

**FIGURE 1:**
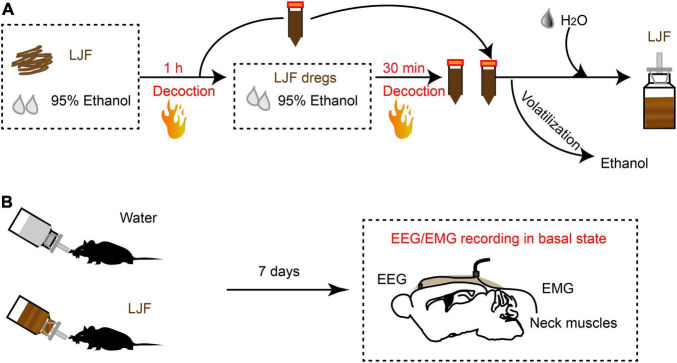
The scheme of LJF extract preparation and EEG-EMG recordings in freely behaving mice. **(A)** Schematic representation shows LJF extract preparation. **(B)** Polysomnography recordings show wakefulness and sleep states in freely behaving mice. LJF, *Lonicerae Japonicae* Flos; EEG, electroencephalogram; EMG, electromyogram.

*Lonicerae Japonicae* Flos was purchased from a Chinese herbal medicine shop (Hengmingtai pharmacy, Xinxiang, Henan, China). LJF (4 g) was heat refluxed for 1 h with 60 ml of 95% ethanol. The filtrate was separated from the residue and stored. The residue was further re-extracted with 95% ethanol for 30 min. The filtrate from the second extraction was collected and mixed with the filtrate from the first batch. The combined filtrate was concentrated with a rotary evaporator to remove ethanol. The final LJF extract was diluted with sterile distilled water to obtain a working solution with a concentration of 4 g/L (Figure 1A).

### Immunofluorescence Staining

The mice were perfused with 50 ml 0.01 M PBS followed by 50 ml 4% paraformaldehyde (PFA). The brains were surgically removed and fixed further with 4% PFA at 4°C for 8–12 h. The vibratome (Leica VT1200S, Germany) was used to cut 40 μm thick brain coronal sections. The brain slices were blocked with 0.01 M PBS solution containing 0.3% Triton X-100 and 10% normal goat serum (NGS, Bosterbio, United States). Then, the sections were incubated overnight at 4°C with rabbit anti-Iba1 antibodies (1: 1,500; Cat. No. 019-19741; Wako Chemicals, Japan). The sections were then washed three times for 15 min each with 0.01 M PBS and incubated with Alexa Fluor 594-conjugated anti-rabbit IgG antibody (1:1,000, Cat. No. A-11012, Invitrogen, United States) for 90 min at room temperature (RT). The sections were then washed three times for 15 min each in 0.01 M PBS followed by incubation with DAPI (5 μg/ml, Cat. No. 10236276001; Roche, Germany) for 15 min. Finally, the sections were washed with 0.01 M PBS and then mounted on a coverslip with medium including 50% glycerol.

### Stereotaxic Surgery and EEG/EMG Electrode Implantation

The mice were administered an intraperitoneal injection (i.p.) of ketamine (100 mg/kg body weight) and xylazine (10 mg/kg body weight), placed in a stereotaxic instrument (RWD Life Science, Shenzhen, China), and implanted with EEG-EMG electrodes (EEG recording electrode: *AP* = 1.75 mm, *ML* = −0.4 mm, EEG reference electrode: cerebellum, EMG electrodes: bilateral neck muscles) as described previously (Hua et al., 2018). The electrodes were cemented with self-curing resins (Super-Bond C&B and dental acrylic). The mice were allowed to recover for 1 week before further experiments.

### Polysomnographic Recording and Analysis

The mice were first acquainted for 3 days with the polysomnography recording set-up by connecting daily to the flexible cables. The EEG and EMG signals were recorded with a Microelectrode AC Amplifier Model 1700 (A-M System, Carlsborg, WA, United States) that was digitized at 250 Hz with the Intracept Chart software in a sound-attenuation box. The signals related to wakefulness, NREM sleep, and REM sleep were recorded manually offline and were defined as previously reported (Hua et al., 2018). Wakefulness was defined by low-amplitude and desynchronized EEG and high EMG activity; NREM sleep was defined by high-amplitude and high power of the EEG delta wave (0.5–4 Hz) and lower EMG activity compared to wakefulness; REM sleep was defined by high power of the EEG theta wave (4–8 Hz) and low EMG activity. The EEG power spectral density was analyzed using the custom-written MATLAB program. EEG power in the 0.5 Hz frequency bins was calculated within the power range of 0.5–80 Hz across time.

### Sleep Deprivation Procedure

The sleep deprivation device (XR-XS108, Shanghai XinRuan Information Technology Co., Ltd., Shanghai, China) for mice consisted of a cylinder with an inbuilt interfering rod. Mice can freely move in a cylinder without food and water restriction. Mice were sleep deprived over a period of 3 h from ZT0 during the day with an interference bar, which was rotated at a speed of 10 rpm every 5 min.

### Sleep Responses to Lipopolysaccharide

Thirty-two hours after sleep deprivation and 1 h before initiation of the dark (active) phase, the mice were administered intra-peritoneal injections of saline (0.1 ml/10 g) or bacterial lipopolysaccharides (10 μg/kg LPS in filtered saline; *Escherichia coli* serotype 0127:B8, Sigma, United States). The EEG and EMG signals were continuously recorded from the beginning of the night.

### Quantitative Real-Time Polymerase Chain Reaction

Total RNA samples were prepared from the medial prefrontal cortex (mPFC) and hippocampus tissue samples using RNAiso Plus (Cat. No. 9109; TaKaRa, Japan). Reverse transcription was performed using the PrimeScriptRT™ Master Mix (Takara, RR036A, Japan) according to the manufacturer’s instructions. Quantitative real-time PCR (1–10 ng cDNA per sample) was performed using the TB Green^®^ Premix Ex Taq™ II (Tli RNaseH Plus; Cat. No. RR820A; TaKaRa, Japan) in the Applied Bio-Systems 7500 system. The relative expression levels of the target mRNAs were calculated using the 2^–ΔΔCT^ method with β-actin as the internal control. Each sample was run in triplicate. The qPCR primers (Lou et al., 2019) used in this study were synthesized by Sangon Biotech, China and are shown in Table 1.

**TABLE 1:** Primers for real-time PCR.

Gene name	Accession number	Forward primer (5′–3′)	Reverse primer (5′–3′)	Product (bp)
IL-6	NM_031168.2	GCCTTCTTGGGACTGATGCT	CTGCAAGTGCATCATCGTTGT	217
TNF-α	NM_013693.3	TCTTCTCATTCCTGCTTGTGG	GGTCTGGGCCATAGAACTGA	128
IL-1β	NM_008361.4	AAGCCTCGTGCTGTCGGACC	TGAGGCCCAAGGCCACAGGT	140
β-actin	NM_007393.5	TGCGTGACATCAAAGAGAAG	TCCATACCCAAGAAGGAAGG	188

### Determination of IL-6, TNF-α, and IL-1β Levels in Plasma by Enzyme Linked Immunosorbent Assay

The mice were anesthetized at 3 h after LPS injection. The whole blood samples were collected and centrifuged at 3,500 rpm and 4^°^C for 10 min. The supernatant was collected for analyzing the levels of serum cytokines using the following commercial enzyme linked immunosorbent assay (ELISA) kits according to the manufacturer’s directions: Mouse IL-6 Ready-SET-Go^®^ ELISA kit (Cat. No. 88-7064; eBiosciences, United States), Mouse IL-1 beta ELISA kit (Cat. No. KE10003; Proteintech, China) and Mouse TNF-alpha ELISA kit (Cat. No. KE10002; Proteintech, China). The absorbance of the samples was detected at 450 nm in the Multiskan FC microplate reader (Thermo Fisher Scienfic, Inc., Shanghai, China).

### Confocal Microscopy Imaging

Immunofluorescence images of the brain sections were captured using the Nikon Ti2-E confocal microscope (Nikon Ti2-e, Japan) with a 10 × Plan Apochromat air objective, 20 × Plan Apochromat air objective, and two laser wavelengths (405 and 561 nm). Image processing and quantitative analysis was performed using ImageJ software.

### Statistical Analysis

Statistical analysis was performed using the SPSS software (v.22, IBM, New York, NY, United States). The experimental data are represented as means ± SEM. Two-tailed Student’s *t*-test was used in comparison of control and LJF group. For LPS administration and LJF treatment statistical analysis were performed using one-way analysis of variance (ANOVA). Statistical significance was set at *P* < 0.05 (^∗^), *P* < 0.01 (^∗∗^), and *P* < 0.001 (^∗∗∗^).

## Results

### *Lonicerae Japonicae* Flos Significantly Increases Non-rapid Eye Movement Sleep in Mice During the Basal Inactive Period

**FIGURE 2:**
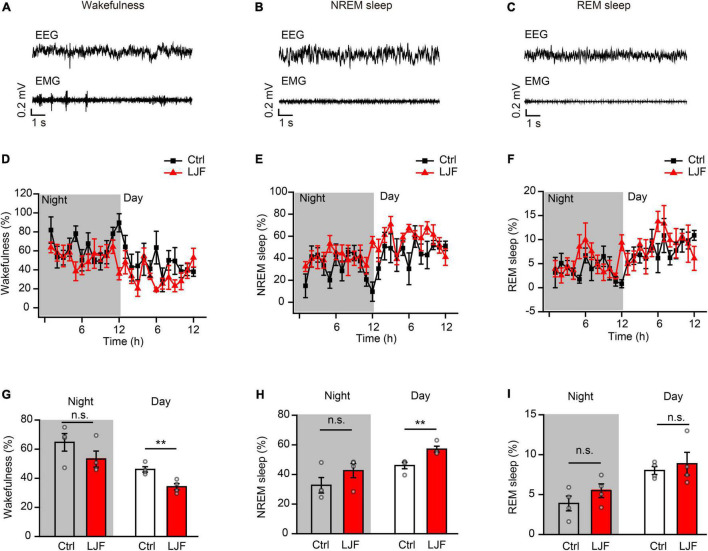
*Lonicerae Japonicae* Flos (LJF) increases sleep and decreases wakefulness in the basal state. **(A–C)** Representative EEG and EMG traces during **(A)** wakefulness, **(B)** NREM sleep, and **(C)** REM sleep in the basal state. **(D–F)** The percentage of time spent in **(D)** wakefulness, **(E)** NREM sleep, and **(F)** REM sleep during night and day in control (Ctrl) and LJF-treated mice. The effects were studied after LJF was administered for 7 days. **(G–I)** Histogram plots show the time spent by the control and LJF treated mice in wakefulness, NREM sleep and REM sleep states during day and night. Each gray dot represents one mouse. The data are expressed as means ± S.E.M. and analyzed by two-tailed unpaired *t*-test. **P < 0.01; n.s., no significance.

To investigate the sleep regulation of LJF in basal state, we firstly implanted electroencephalogram-electromyogram (EEG–EMG) electrodes for polysomnography (Figure 1B). The EEG and EMG waves were analyzed during wakefulness, non-rapid eye movement (NREM) sleep, and rapid eye movement (REM) sleep (Figures 2A–C). After recovery, the mice were allowed to have *ad libitum* access to water containing LJF for 7 consecutive days (Figure 1B). Then, the effects of LJF were analyzed by quantifying the time spent in wakefulness, NREM sleep, and REM sleep during day and night in the control and LJF-treated mice. LJF-treated mice showed reduced time spent in wakefulness (*P* < 0.05) and increased time spent in NREM sleep during the day (*P* < 0.05) compared to the control group (Figures 2D–F). However, the time spent at night in wakefulness, NREM sleep, and REM sleep was similar between the two groups of mice (Figures 2G–I). The EEG power spectrum was analyzed during NREM sleep at day and night. The EEG results did not show any statistically significant differences in the power of the delta and gamma bands between the control and LJF-treated mice (Supplementary Figure 1). These results demonstrated that LJF had sleep-promoting effects during the inactive period without affecting sleep pressure.

### *Lonicerae Japonicae* Flos Promotes Homeostatic Sleep After Sleep Deprivation

**FIGURE 3:**
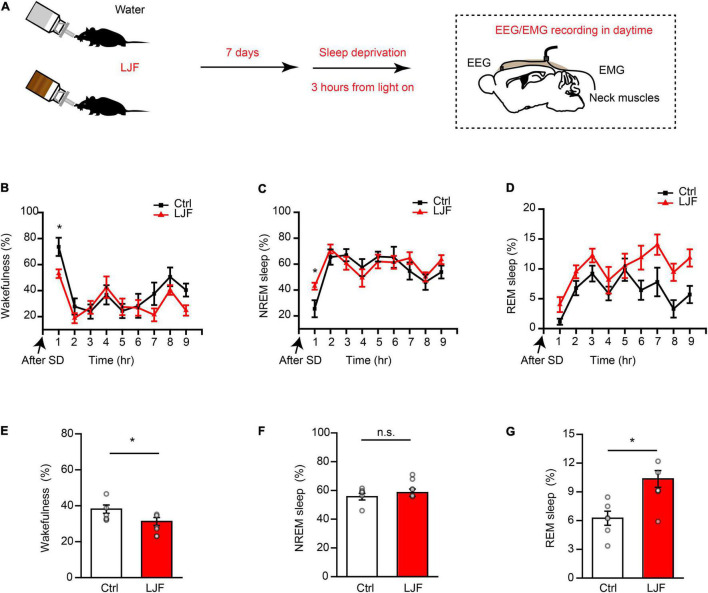
*Lonicerae Japonicae* Flos (LJF) promotes homeostatic sleep in mice subjected to acute sleep deprivation. **(A)** The scheme of sleep recordings to analyze the effects of LJF in acute sleep-deprived mice. **(B–D)** Time spent by the control and LJF-treated mice in **(B)** wakefulness, **(C)** NREM sleep, and **(D)** REM sleep after 3 h sleep deprivation. **(E–G)** Histogram plots show the time spent by control and LJF-treated mice in **(E)** wakefulness, **(F)** NREM sleep, and **(G)** REM sleep over a period of 9 h after sleep deprivation. Each gray dot represents a single mouse. The data are expressed as means ± S.E.M. *n* = 6 per group; two-tailed unpaired *t*-test was used to compare the data; *P < 0.05; n.s., no significance.

Sleep deprivation (SD) is a serious health problem for the adult population worldwide. Therefore, we investigated the effects of LJF on homeostatic sleep by performing SD beginning at zeitgeber time (ZT) 0 to mimic prolonged wakefulness (Figure 3A). Then, we recorded the EEG and EMG signals during the day (Figures 3B–D). The time spent in wakefulness was significantly lower (*P* < 0.05) (Figures 3B,E), and the time spent in REM sleep during recovery was significantly increased (*P* < 0.05) in the LJF-treated mice compared to the control mice (Figures 3D,G). Moreover, the time spent in NREM sleep was higher in the LJF-treated mice compared to the control mice, but was statistically insignificant (Figures 3C,F).

**FIGURE 4:**
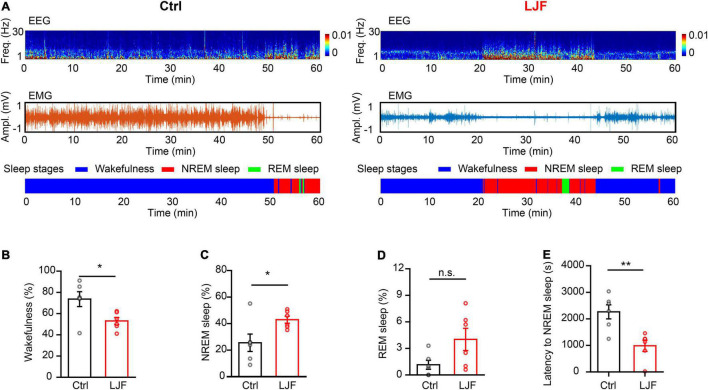
*Lonicerae Japonicae* Flos (LJF) promotes sleep recovery after acute sleep deprivation. **(A)** The representative spectrograms display interchange of brain states in the first hour after sleep deprivation between the control (left) and LJF-treated (right) mice. **(B–E)** Time spent by the control and LJF-treated mice in (B) wakefulness, **(C)** NREM sleep, **(D)** REM sleep, and **(E)** latency to NREM sleep after acute sleep deprivation in the first hour. *n* = 6 per group; The data are expressed as mean ± S.E.M; two-tailed unpaired *t*-test was used for comparing data; **P < 0.01, *P < 0.05. n.s., no significance.

The first hour of sleep recovery represents the most significant stage of the sleep cycle. Therefore, we examined the time course of wakefulness, NREM sleep, and REM sleep as well as EEG power in the control and LJF-treated mice (Figure 4A). The time spent in wakefulness was significantly reduced (*P* < 0.05) and the time spent in NREM sleep was significantly increased (*P* < 0.05) in the LJF-treated mice compared to the controls (Figures 4B,C). Furthermore, the time spent in REM sleep was increased in the LJF-treated mice compared to the control mice but was not statistically significant (Figure 4D). Moreover, the latency to NREM sleep was significantly decreased in the LJF-treated mice compared to the control mice (*P* < 0.01) (Figure 4E). This demonstrated that LJF significantly increased sleep. Next, we analyzed the EEG power spectra during NREM sleep in the first hour after SD (Supplementary Figure 2A) and did not find any differences in the delta and gamma bands between the control and LJF-treated mice (Supplementary Figure 2B). These results demonstrated that LJF increased homeostatic sleep in mice subjected to acute sleep deprivation.

### *Lonicerae Japonicae* Flos Modulates Sleep-Wake Patterns After Lipopolysaccharide Administration

**FIGURE 5:**
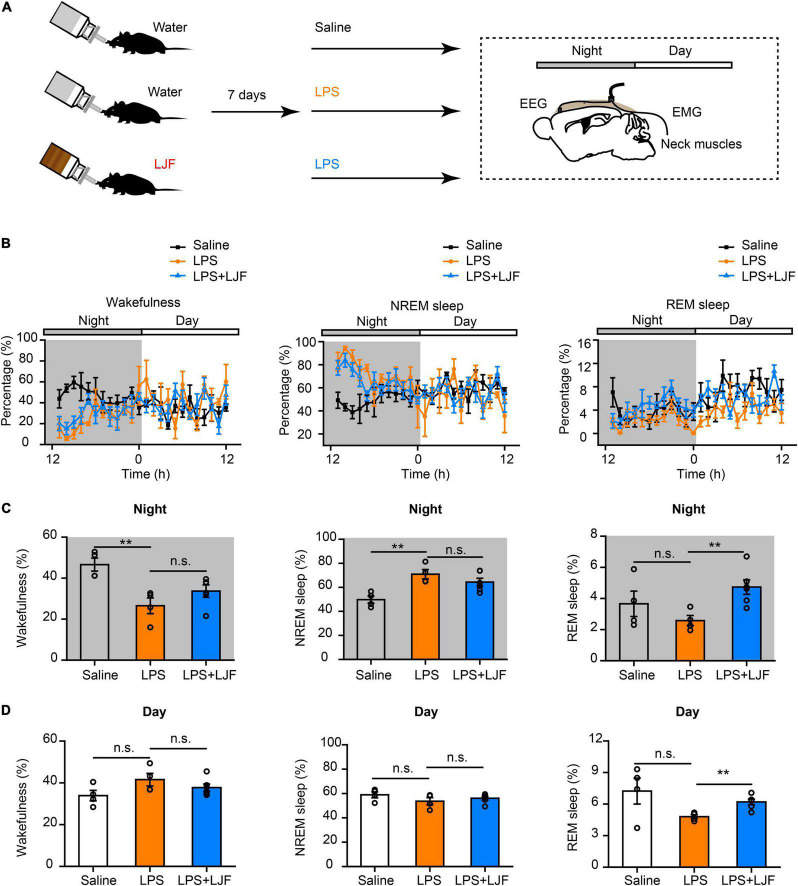
*Lonicerae Japonicae* Flos (LJF) promotes sleep in the LPS- challenged mice. **(A)** The scheme of sleep recordings after LPS administration in the control and LJF-treated mice. **(B)** Time spent in wakefulness (left), NREM sleep (middle), and REM sleep (right) by the saline (*n* = 4), LPS (*n* = 4), and LPS + LJF (*n* = 6) groups of mice. **(C,D)** Histogram plots show the time spent in wakefulness (left), NREM sleep (middle), and REM sleep (right) during **(C)** night and **(D)** day by the saline (*n* = 4), LPS (*n* = 4), and LPS + LJF (*n* = 6) groups of mice. Each dot represents one mouse. Data are expressed as means ± S.E.M. The differences between groups were assessed by one-way ANOVA followed by LSD *post-hoc* test. **P < 0.01; n.s., no significance.

Lipopolysaccharide (LPS) plays a key role in the crosstalk between immunity and sleep (Zielinski et al., 2017; Christian et al., 2018; Statello et al., 2019). In mice, a single dose of LPS elicited immune responses that altered the sleep-wake cycle (Surbhi et al., 2019; Klawonn et al., 2021). Therefore, we injected the control and LJF-treated mice with a single dose of LPS (10 μg/kg) at ZT 11 and continuously recorded their EEG and EMG signals for 24 h from ZT12 (Figures 5A,B). LPS significantly reduced the time spent in wakefulness (*P* < 0.01) and increased the time spent in NREM sleep (*P* < 0.01) during the night but did not show any effects during the day (Figures 5C,D). LJF administration increased the time spent in REM sleep during both day (*P* < 0.01) and night (*P* < 0.01) and reversed the effects of LPS on wakefulness and NREM sleep (Figures 5C,D).

Delta (1–4 Hz) and gamma (25–80 Hz) waves are typically associated with sleep pressure and/or exploratory wakefulness. Therefore, we analyzed spectral power of the delta and gamma waves to determine differences in EEG power during NREM sleep (Supplementary Figures 3A,C). The power spectra of delta waves did not show significant differences between the saline-, LPS- and LPS + LJF-treated mice. However, power spectra of the gamma waves were significantly lower in the LJF-treated mice compared the LPS group (*P* < 0.05; Supplementary Figures 3B,D). This suggested that EEG activity was reduced as an adaptation to maintain sleep homeostasis. Moreover, these results demonstrated that LJF increased the time spent in REM sleep in response to LPS-induced inflammation.

### *Lonicerae Japonicae* Flos Inhibits Lipopolysaccharide-Induced Microglial Activation and Reduces the Levels of Proinflammatory Cytokines in the Blood and Brain Tissues

**FIGURE 6:**
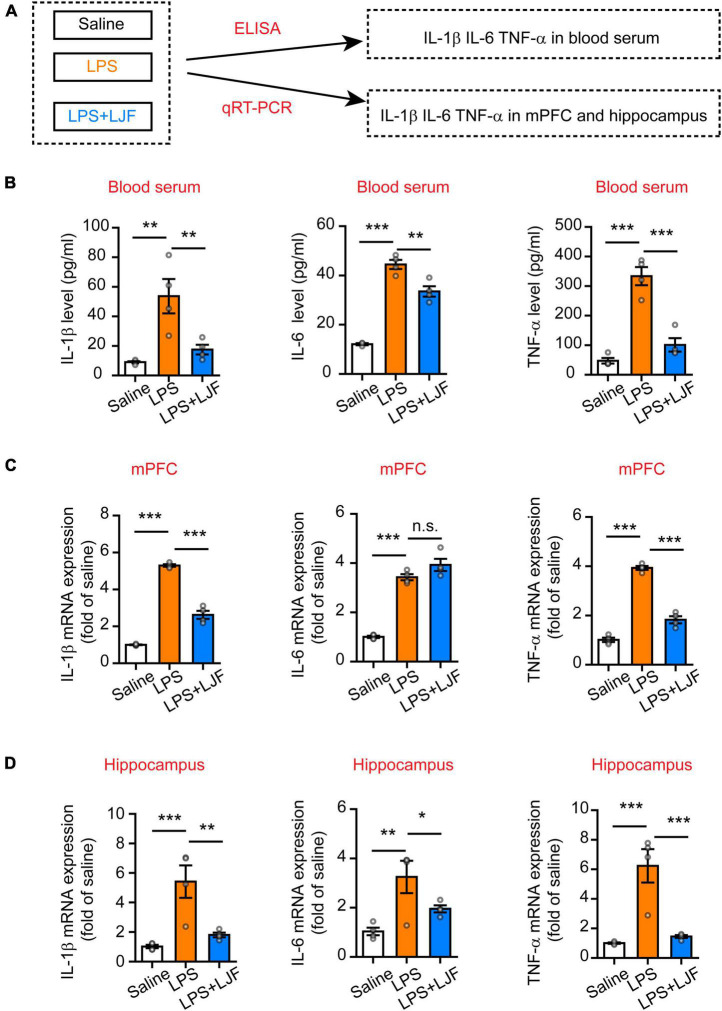
*Lonicerae Japonicae* Flos (LJF) decreases the levels of proinflammatory cytokines in the blood serum and brain tissues of LPS- challenged mice. **(A)** The scheme of analyzing the effects of LJF on LPS-induced inflammation by estimating the levels of pro-inflammatory cytokines in blood and brain tissues of saline, LPS-treated, and LPS + LJF-treated groups of mice. **(B–D)** The histogram plots showing the levels of pro-inflammatory cytokines, namely, IL-6, TNF-α, and IL-1β in the **(B)** blood serum, **(C)** mPFC, and **(D)** hippocampus. The cytokines were estimated by ELISA in the blood serum and by quantitative real-time polymerase chain reaction (qRT-PCR) in the mPFC and hippocampus tissues. *n* = 3 or 4 per group. The data are expressed as means ± S.E.M. Each dot represents one mouse. The differences between the groups were analyzed by one-way ANOVA followed by LSD *post-hoc* test; *P < 0.05, **P < 0.01, ***P < 0.001. n.s., no significance.

The causal link between inflammation and sleep is well-established in the animal models (Zielinski et al., 2013; Atrooz and Salim, 2020). The medial prefrontal cortex (mPFC) regulates both wake-sleep cycles (Lou et al., 2020) and inflammation (Zhang et al., 2019). Hippocampus plays a critical role in regulating mood, cognition, memory, inflammation, and sleep (Wan et al., 2007; Brankack et al., 2009). Therefore, we analyzed the effects of LJF on LPS-induced inflammation and REM sleep by estimating the levels of proinflammatory cytokines, such as, interleukin (IL)-6, IL-1β, and tumor necrosis factor α (TNF-α) in the blood plasma, hippocampus, and mPFC (Figure 6A). LPS significantly increased IL-1β (*P* < 0.05), IL-6 (*P* < 0.05), and TNF-α (*P* < 0.05) levels in the blood serum, mPFC, and hippocampus (Figures 6B–D). However, LJF-treated mice showed significantly reduced levels of IL-1β (*P* < 0.05), IL-6 (*P* < 0.05), and TNF-α (*P* < 0.05) compared to the LPS-challenged mice in the blood serum, mPFC, and hippocampus (Figures 6B–D). These results demonstrated that LPS increased the levels of inflammatory cytokines in the plasma and the brain. However, LJF suppressed LPS-induced microglial activation, thereby reducing the levels of pro-inflammatory cytokines in the blood and brain of mice.

**FIGURE 7:**
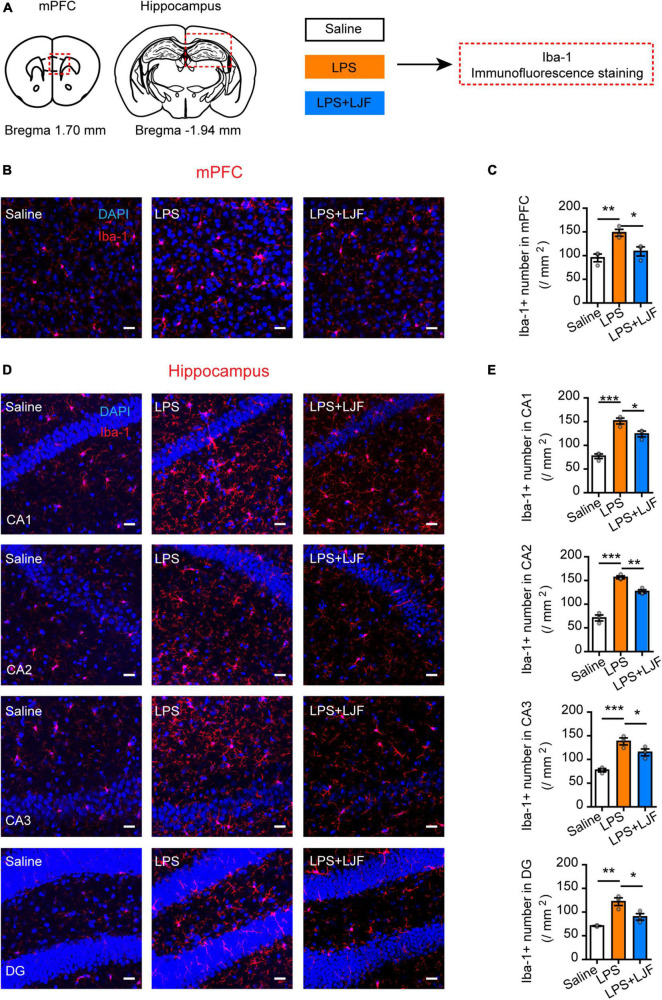
*Lonicerae Japonicae* Flos (LJF) inhibits microglial activation in the mPFC and hippocampus tissues of LPS- challenged mice. **(A)** The scheme of assessing the effects of LFJ on microglial activation in LPS-treated mice. **(B,D)** The representative images show the expression of Iba-1 in **(B)** mPFC and **(D)** hippocampus tissues of saline, LPS-treated, and LJF + LPS treated mice (*n* = 3–4). Scale bar, 25 μm. **(C,E)** The histogram plots showing the numbers of Iba1^+^ cells in the **(C)** mPFC and **(E)** hippocampus regions of the brains in the saline, LPS-challenged, and LJF + LPS treated mice (*n* = 3 per group). The data are expressed as means ± S.E.M. Each dot represents one mouse. The differences between groups were compared using one-way ANOVA followed by LSD *post-hoc* test. *P < 0.05, **P < 0.01, *** *P* < 0.001.

We then examined if LJF suppressed the activation of microglia by LPS in the mPFC and hippocampus regions of the brain. The expression of Iba-1 (ionized calcium binding adaptor molecule 1), which is specifically expressed in the microglia of brain tissues was analyzed by immunofluorescence staining of the brain sections using the Iba-1 antibody (Figure 7A). The results showed that LPS treatment significantly increased the numbers of microglia (Iba-1^+^ cells) in the mPFC, hippocampal CA1, CA2, CA3, and dentate gyrus (DG) regions of the brain (Figures 7B–E). However, the numbers of Iba-1^+^ cells were significantly reduced in the mPFC and hippocampal CA1, CA2, CA3, and DG regions of the LPS + LJF-treated mice compared to the LPS-treated mice (Figures 7B–E). These data suggested that LJF reduced neuro-inflammation by inhibiting LPS-induced microglial activation.

## Discussion

Since Youyou Tu won the Nobel Prize for the discovery of artemisinin and its application in disease treatment, TCM and herbal extracts, particularly bioactive ingredients, have attracted increasing attention. LJF is commonly used in TCM for thousands of years. The polysomnography data showed that LJF increased sleep during the inactive state. In the sleep-deprived mice, LJF increased the time spent in REM sleep for recovery. In the LPS-challenged mice, LJF increased time spent in REM sleep and inhibited microglial activation. Together, these findings suggest that LJF may alleviate sleep disorders associated with sleep disturbance and LPS-induced inflammation.

Previous studies have suggested that sleep and wakefulness states are regulated by a two-process model, which includes the circadian system and mechanisms associated with sleep homeostasis (Borbely and Achermann, 1999). Sleep loss is the most sleep problem. Especially with the overuse of electronic products and the changes of human sleep habits, people have more and more sleep problems, which brings a heavy burden to the individuals and society. China has a long history to treat insomnia by using TCM and specific treatment such as bath therapy. The active ingredient of TCM can be absorbed through the skin to assist in the treatment of sleep disorders (Liao et al., 2008; Ni et al., 2015; Haghayegh et al., 2019; Aghamohammadi et al., 2020). According to the analysis of clinical data, the potential pharmacological effects have been found in TCM for treating insomnia through the regulation of neurotransmitters and their receptors in the brain, including γ- aminobutyric acid, serotonin, orexin, acetylcholine (Shi et al., 2014; Singh and Zhao, 2017).

It has been found that the main components of LJF are chlorogenic acid and luteolin. Chlorogenic acid, one of the most abundant effective acids, naturally exists in coffee and tea extract, and has sleep regulation and neuroprotective effects (Heitman and Ingram, 2017; Kawada, 2018; Naveed et al., 2018). Luteolin, a universal flavonoid, has a sleep-promoting effect though adenosine receptors (Kim et al., 2019). However, the composition of the active ingredients of LJF vary according to the extraction methods. In the relative effectiveness of suppressing NO formation, the inhibitory ability of ethanol extract of LJF was superior to that of water extract. Meanwhile, water extract of LJF showed higher antioxidant activities (Hsu et al., 2016). We will be interested in the components of active compounds in two different extracts and their roles in sleep regulation. In the future, preclinical and clinical studies are necessary to determine the roles of specific active components of the LJF extract in sleep homeostasis and their effects on the neurotransmitters in specific brain regions, which will also widely explore the functions of TCM containing similar active components.

Epidemiological and clinical studies have demonstrated that sleep disturbance induces oxidative stress and inflammation (Irwin et al., 2016). We investigated the regulatory effects of LJF on sleep homeostasis in response to sleep deprivation and LPS-induced inflammation. Microglial cells are the primary innate immune cells in the brain that play a critical role in sleep-wakefulness and neuro-inflammation (Deurveilher et al., 2021). Previous studies have reported that neuro-inflammation associated alterations in the mPFC and hippocampus regions of the brain (Atrooz et al., 2019; Chang et al., 2021). The mPFC region is activated by stress-induced sleep-wake disturbances (Bellesi et al., 2017). Moreover, dysfunctional mPFC circuit is associated with hyperarousal symptoms (Lou et al., 2020). LJF protects against cognitive dysfunction by decreasing neuro-inflammation in the hippocampus (Shao et al., 2020). Furthermore, hippocampal neuronal plasticity is intricately associated with NREM sleep regulation (Brankack et al., 2009; Levenstein et al., 2019). Therefore, in this study, we focused on the relationship between anti-inflammatory effects of LJF and sleep homeostasis in relation to the mPFC and hippocampus regions of the brain, thereby establishing the link between immunity and sleep. Our findings demonstrated that LJF suppressed LPS-induced inflammation in specific brain regions related to sleep. Further studies are necessary to investigate the biochemical mechanisms underlying the effects of LJF including the modulation of the neural circuit mechanisms in specific brain regions that regulate sleep homeostasis.

In summary, our results showed that LJF regulated wakefulness and sleep structure under basal conditions, as well as in response to sleep deprivation and LPS-induced sleep disorder. Therefore, our study demonstrated the potential clinical value of LJF in alleviating sleep disturbance associated with sleep pressure and inflammation. However, further preclinical and clinical studies are necessary to confirm our findings in human subjects.

## Data Availability Statement

The original contributions presented in the study are included in the article/Supplementary Material, further inquiries can be directed to the corresponding authors.

## Ethics Statement

The animal study was reviewed and approved by the Animal Experimentation Ethics Committee of the Xinxiang Medical University, China.

## Author Contributions

HW and RH designed and supervised the study and wrote the manuscript with feedback from all authors. YD and KL performed most of the experiments. XL and BN performed the extract of LJF. XC wrote the MATLAB code to analyze EEG/EMG data. RH and JZ performed histology, immunostaining, confocal imaging, and analyzed the immunofluorescence and EEG/EMG data. XZ and JX provided the experimental equipment and technical assistance. All authors contributed to the article and approved the submitted version.

## Conflict of Interest

The authors declare that the research was conducted in the absence of any commercial or financial relationships that could be construed as a potential conflict of interest.

## Publisher’s Note

All claims expressed in this article are solely those of the authors and do not necessarily represent those of their affiliated organizations, or those of the publisher, the editors and the reviewers. Any product that may be evaluated in this article, or claim that may be made by its manufacturer, is not guaranteed or endorsed by the publisher.
