# Supplementary material for: Lonicerae Japonicae Flos Extract Promotes Sleep in Sleep-Deprived and Lipopolysaccharide-Challenged Mice
Source: Front Neurosci. 2022 Apr 12;16:848588. doi: 10.3389/fnins.2022.848588 (PMC9040552; doi:10.3389/fnins.2022.848588)
Supplement: Supplementary file 1 [file Data_Sheet_1.docx]

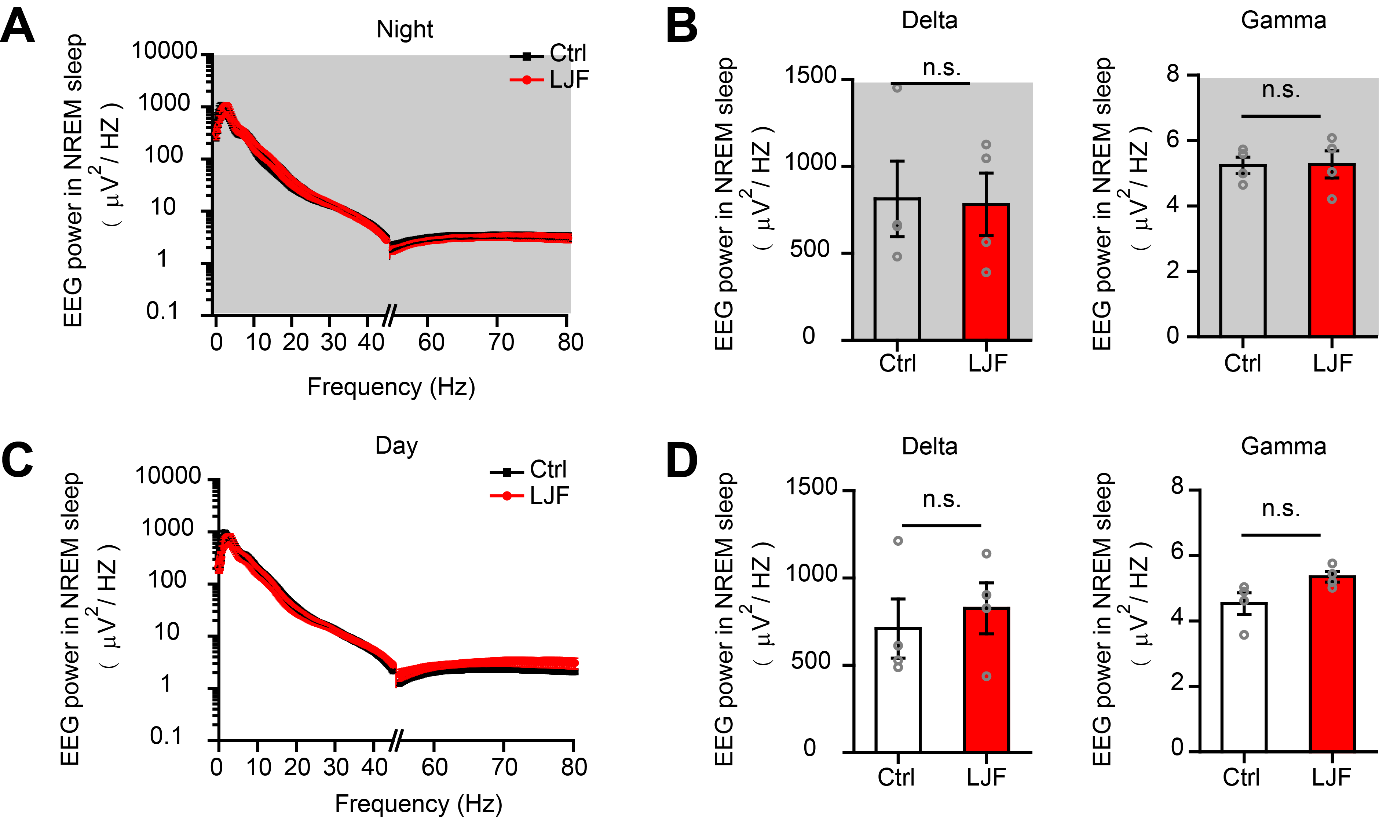


**Supplementary Figure 1. LJFhasnoeffect on EEG power spectrumin the basal state.** (A and C) NREM sleep EEG power spectrum on nighttime(A) and daytime(C) between control(water) and LJF-treated mice. LJF has no significance on NREM sleep EEG power over 0.5-80 Hz frequency ranges. (B and D) Statistical chart of NREM sleep EEG power spectrum on nighttime(B) and daytime(D) after LJF and water. Note: n=4 pre group. Data are expressed as means ± S.E.M. Two-tailed unpaired *t*-test. n.s., no significance.


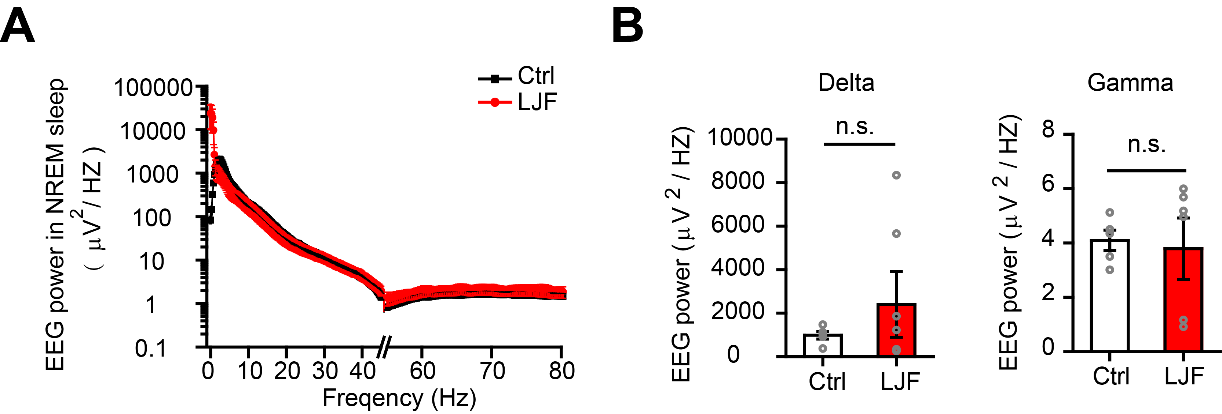


**Supplementary Figure 2. LJF hasfew effect on EEG power spectrum subjected to acute sleep deprivation.** (A) EEG spectral power in NERM sleep after acute sleep deprivation. (B) Statistical chartshowing NERM sleep EEG spectral power of delta (left) and gamma (right) on the first hour of sleep recovery. EEG spectral power had no significance between control(water) and LJF-treated mice.Note: n=6 per group. Data are expressed as means ± S.E.M. Two-tailed unpaired *t*-test. n.s., no significance.


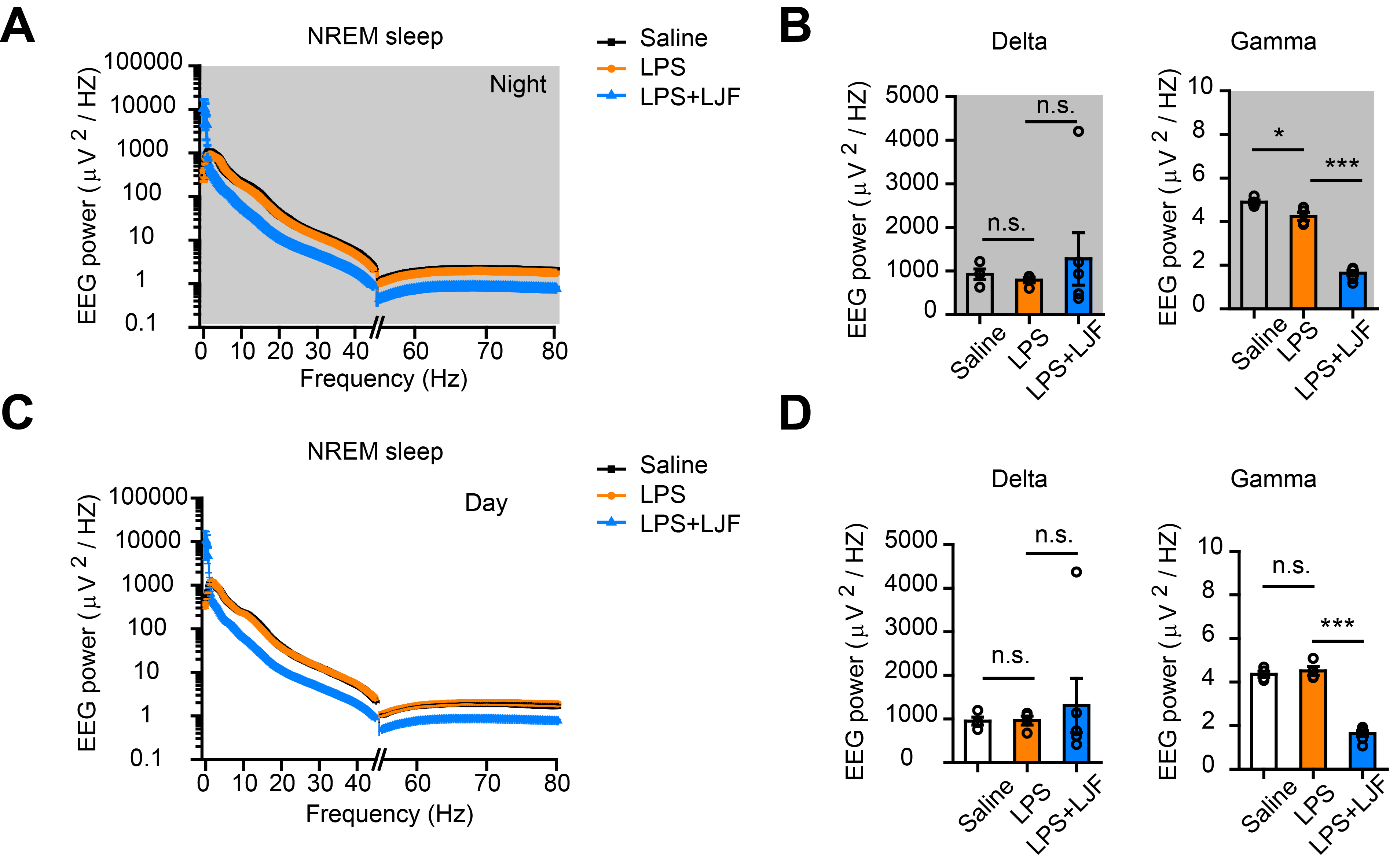


**Supplementary Figure 3.LJF hasfew effect on EEG power spectrum afterLPS.**(A and C) NERM sleep EEG spectral power on nighttime (A) and daytime (C)in the saline (n=4), LPS (n=4), and LPS+LJF (n=6) mice. (B and D) Histogram plots showing NERM sleep EEG spectral power of delta (left) and gamma (right) on the nighttime (B) and daytime (D). LJF reduced EEG spectral power of gamma compared to LJF-challengedmice.Data are expressed as means ± S.E.M. One-way ANOVA followed by LSD post hoc test. **P*< 0.05, ***P* <0.01,****P*< 0.001, n.s., no significance.
